# A nationwide study on the prevalence and contributing factors of obstructive sleep apnea in Iran

**DOI:** 10.1038/s41598-023-44229-w

**Published:** 2023-10-17

**Authors:** Khosro Sadeghniiat-Haghighi, Samaneh Akbarpour, Atefeh Behkar, Rahmatollah Moradzadeh, Zahra Banafsheh Alemohammad, Nazanin Forouzan, Ali Mouseli, Hamed Amirifard, Arezu Najafi

**Affiliations:** 1 Sleep Breathing Disorders Research Center, Tehran University of Medical Sciences, Tehran, Iran; 2 Occupational Sleep Research Center, Baharloo Hospital, Tehran University of Medical Sciences, Tehran, Iran; 3 Department of Epidemiology, School of Health, Arak University of Medical Sciences, Arak, Iran; 4 Department of Health Services Management, Social Determinants in Health Promotion Research Center, Hormozgan Health Institute, Hormozgan University of Medical Sciences, Bandar Abbas, Iran; 5 Iranian Center of Neurological Research, Neuroscience Institute, Tehran University of Medical Sciences, Tehran, Iran

**Keywords:** Epidemiology, Respiratory tract diseases

## Abstract

Reliable obstructive sleep apnea (OSA) prevalence information in Iran is lacking due to inconsistent local study results. To estimate OSA prevalence and identify clinical phenotypes, we conducted a nationally representative study using multi-stage random cluster sampling. We recruited 3198 individuals and extrapolated the results to the entire Iranian population using complex sample survey analyses. We identified 3 clinical phenotypes as “sleepy,” “insomnia,” and “restless legs syndrome (RLS).” The prevalence of OSA was 28.7% (95%CI: 26.8–30.6). The prevalence of “sleepy,” “insomnia,” and “RLS” phenotypes were 82.3%, 77.8%, and 36.5% in women, and 64.8%, 67.5%, and 17.9% in men, respectively. “Sleepy” and “insomnia” phenotypes overlapped the most. Age (OR: 1.9), male sex (OR: 3.8), BMI (OR: 1.13), neck circumference (OR: 1.3), RLS (OR: 2.0), and insomnia (OR: 2.3) were significant OSA predictors (p-values: 0.001). In men, “sleepy” phenotype was associated with youth and unmarried status but not in women. The “insomnia” phenotype was associated with shorter sleep duration in women; cardiovascular diseases (CVD), urban residency, and shorter sleep duration in men. “RLS” phenotype was associated with shorter sleep duration and CVD in women and older age, lower educational level, CVD, and hypertension in men. The findings point to the need for funding of OSA screening in Iran, for a different assessment of men and women, and for future sleep research to consider overlapping phenotypes.

## Introduction

Undiagnosed obstructive sleep apnea (OSA) is annually estimated to cost 149.6 billion dollars, with an additional $3.4 billion in medical costs in the United States^1,2^. According to a large study including 16 countries, OSA affects about 1 billion adults globally between the ages of 30 and 69. Due to the significant prevalence and impact of OSA worldwide, it is more crucial than ever to conduct high-quality epidemiological research, including national surveys, and to establish a global registry^3,4^.

The knowledge of disease burden can aid policymakers in making reasonable decisions. Unfortunately, there are limited local studies on OSA prevalence in Iran with discrepancies in reported prevalence rates; studies carried out in our country indicated different OSA prevalence rates, ranging from 5 to 27%^5,6^. In a global literature-based analysis in 2019, the extrapolated percentages of individuals with moderate and severe OSA in Iran were 41.8% and 24.1%, respectively^4^. These small studies, which lacked a sound methodology and had inconsistent findings, do not accurately reflect Iran's total population. Given that undiagnosed OSA may cause several major health issues, this information gap might have a negative health impact on the people of Iran.

Besides its significant prevalence and effect, OSA is a disorder with diverse clinical phenotypes. The heterogenicity of OSA clinical faces was poorly recognized until recently. A study of clusters on patients with OSA has classified clusters as “disturbed sleep group,” excessive daytime sleepiness group,” and “minimally symptomatic group^7^.” Cardiovascular comorbidity varies among various OSA clinical phenotypes; it was more common in the “insomnia” phenotype and less common in “sleepy” phenotype^8^. Understanding the OSA phenotypes is crucial for the clinical application of precision medicine for OSA because different OSA clinical phenotypes may require different treatments. For example, a “sleepy” phenotype may benefit from psychological therapy or sedative hypnotics, whereas patients with a “RLS” phenotype may benefit from specific RLS treatment^9^. In Iran, the prevalence of OSA phenotypes, particularly overlapping phenotypes, is not well-established^10^.

This study addresses a critical knowledge gap or uncertainty in the prevalence of OSA in Iran, using a robust methodology that represents the diverse population from north to south and east to west. It offers insights into the possible clinical phenotypes of the Iranian population that are at a higher risk for OSA. The results can provide valuable information to healthcare providers and policymakers, ultimately leading to improved health outcomes for the Iranian population.

## Methods

### Study design

This cross-sectional population-based study was conducted in 7 randomly selected provinces of Iran from 2017 to 2020 (see Table S1 in the supplemental material). The high-risk population was screened for OSA using reliable, validated Persian questionnaires and self-reported symptoms. A physical examination was performed by trained personnel. The study protocol complies with the Declaration of Helsinki, and the National Institute for Medical Research Development Ethics Committee approved and funded the study (IR NIMAD REC 1396 102 with a grant number of 957726).

### Sampling method

#### Selecting provinces and health care centers

We randomly included 7 provinces out of 31. The sample size for each province was calculated based on the province’s total population (see Table S1 in the supplemental material). Then, urban and rural health care centers (HCC) were randomly selected in each province to reach the targeted sample size. 75% of the targeted sample size was collected from urban HCCs, whereas 25% was collected from rural HCCs.

After determining province and HCCs, the funding institute (NIMAD) made the necessary arrangements with the vice-chancellors of health in the selected provinces. The provincial deputy then determined a contributor for each province for further collaboration.

The staff was then trained on the study’s goals, objectives, and specifics of using the study instruments, such as collecting data and completing questionnaires. They also received information on sleep health, sleep disorders, OSA, and treatment options from an epidemiologist and a sleep medicine physician who traveled to each province.

#### Selecting the participants

The population of each HCC was sampled using household codes recorded over there. The executive team at the Occupational Sleep Research Center at Baharloo Hospital in Tehran was responsible for selecting the household codes for HCC’s clusters.

Then, in collaboration with the trained health center’s personnel, two professional interviewers visited the selected households and outlined the study's goals and objectives. One male and one female who were at least 18 years old, Iranian, and agreed to participate in the study were selected. Informed consent was obtained from all participants. Interviewers followed the same procedures after moving to the house to the right of the one they had chosen. Using the indicated method, interviewers sampled continuously until each cluster included 20 individuals (10 males, 10 females, 2 in each age group, 18–25, 25–35, 35–45, 45–55, and > 55 years old). If the eligible participant was not available in a household, further follow-up in upcoming days would be performed, or the sampling would be continued until the defined cluster was completed. After completing the surveys, residents received information about OSA, sleep disorders, and treatment options. This information was also provided to anyone who did not consent to participate. At the end of the interview and measurement of anthropometric indices, each household was given a pamphlet with the identity information of the province's in-charge medical university and our research center. The color-illustrated pamphlet featured symptoms, adverse health effects, methods of diagnosis and treatments, and a table for calculating an individual's risk of OSA based on the STOP-BANG score (see Fig. S1 in the supplemental material).

We conducted two quality assurance tests. The first quality check was performed by HCC administrators after the questionnaires were completed, and the second quality check was performed by the executive manager of the study after the questionnaires were sent to the research center. They ensured that each cluster had an equal distribution of males and females, and included two participants from each age group.

### Sample size

With a prevalence of variables with proportions at least %35, for a 95% confidence interval and precision of 0.05 *(Prevalence considered as 35%), and considering a design effect of 1.5 and a response rate of 85%, the total calculated sample size was: n = x × 1.5 × 1/0.85 = 5034 which was rounded to 5100^11^.

### Study measures

After obtaining informed consent, participants completed demographic, STOP-BANG, Insomnia Severity Index (ISI), IRLSSG questionnaires, and several questions on their sleeping habits.

A popular OSA screening method is the STOP-BANG questionnaire. The STOP questionnaire measures snoring, daytime fatigue, observed apnea, and elevated blood pressure^12^. The STOP-BANG questionnaire combines the aforementioned items with BMI, Age, Neck circumference (NC), and Gender^13^; STOP-BANG score ≥ 3 indicates a high risk for OSA with high sensitivity. We used the Persian version of the STOP-BANG questionnaire validated by Sadeghniiat et al.^14^ (sensitivity of 91.1 and specificity of 45.2 at AHI level ≥ 5).

Insomnia Severity Index (ISI) is a 7-item self-report questionnaire evaluating insomnia^15,16^. A total ISI score of ≥ 8 is defined as insomnia. We used the validated Persian version of ISI by Yazdi et al.^17^.

International Restless Legs Syndrome Study Group (IRLSSG) has introduced a self-reported, validated rating scale for diagnosing and assessing RLS^18^. It comprises four questions. If a patient replied “yes” to each of the four IRLSSG questions, it was considered they experienced RLS.

To calculate sleep duration, the 4th question of the Persian version of the Pittsburgh Sleep Quality Index (PSQI) questionnaire by Farahi et al.^19^ was included (During the past month, how many hours of actual sleep did you get at night? (This may differ from the number of hours you spend in bed)).

We measured participants’ weight (PS 502 GR, MATHEO, Germany), height (NTT9650, NOVA, China), and NC (cloth measuring tape) using the same brand of weighing scales and measurement tapes.

### Clinical phenotype determination of patients with a high risk for OSA

We defined three phenotypes, including “sleepy,” “insomnia,” and “RLS”. The “sleepy” phenotype was characterized as a positive response to “Do you feel sleepiness or fatigue during the day,” adapted from the STOP-BANG questionnaire. The “insomnia” phenotype was characterized by an ISI score of more than 7. “RLS” phenotype was characterized as answering yes to all four RLS questionnaire items mentioned before.

### Statistical analysis

STATA software was used for all statistical analyses. The Kolmogorov–Smirnov test was used to assess the normality of quantitative variables before data analysis. Complex sample survey analyses were performed to extrapolate the results to the Iranian adult population. The weight was based on the 2016 national Iranian census to match the age (10-year strata), sex, and area of residence (rural/urban).

The prevalence of OSA and its confidence interval were estimated. To evaluate the two groups, an independent student’s t-test was utilized. If there were more than two groups, a one-way ANOVA was conducted to compare the groups. Chi-square tests were utilized for categorical variables. Finally, a multivariate logistic regression analysis was performed to estimate the association between OSA and various independent variables. For statistical analysis, quantitative missing data were handled via mean substitution, and qualitative missing data were handled via removal. *p*-values of less than 0.05 were considered statistically significant.

### Ethics approval and consent to participate

The study protocol complies with the Declaration of Helsinki and the Ethics Committee of the National Institute for Medical Research Development approved the study (IR NIMAD REC 1396 102). Informed consent was obtained from all participants.

## Results

We included 3198 participants (1599 (50%) male) with a mean age of 39.7 (95%CI: 39.6–39.8) years. Most participants were married (70.8%). The study population had a mean body mass index (BMI) of 26.5 (95%CI: 26.1–26.9) kg/m^2^ and a mean NC of 37.6 (95%CI: 37.3–37.9) cm. The mean sleep duration was 7 (95%CI: 7–7.3) hours/night. Table 1 presents baseline demographic characteristics of the participants.

**Table 1:** Contributing factors of OSA among study participants.

Variable	Total	Non-OSA	OSA	*p*-value
Demographic				
Age, mean (95%CI), years	39.7 (39.6–39.8)	35.4 (35.0–35.8)	50.8 (50.0–51.7)	0.001
Male	50.0 (50.0–50.0)	42.1 (41.0–43.3)	69.7 (68.2–71.1)	0.001
Marital status				0.001
Single	23.8 (21.6–26.0)	28.9 (25.4–32.6)	11.0 (8.6–14.1)	
Married	70.8 (68.3–73.2)	67.2 (63.1–71.0)	80.0 (76.8–82.8)	
Divorced	1.7 (1.0–2.8)	1.6 (1.0–2.5)	1.9 (1.0–3.5)	
Widow	3.8 (3.2–4.5)	2.4 (2.1–2.9)	7.1 (5.8–8.7)	
Education				0.001
Illiterate	4.8 (2.7–8.4)	2.3 (1.2–4.5)	11.0 (6.1–18.9)	
Nehzat^a^	1.8 (1.3–2.5)	1.0 (0.6–1.5)	3.8 (2.4–5.8)	
Primary school	19.0 (16.8–21.4)	16.1 (13.8–18.5)	26.3 (23.1–29.7)	
Highschool/Diploma	38.0 (35.5–40.5)	41.1 (38.4–43.8)	30.2 (27.4–33.1)	
Associate degree/bachelor	30.1 (27.5–32.9)	32.6 (29.6–35.8)	24.0 (20.2–28.3)	
Master/doctorate	6.4 (5.1–7.8)	6.9 (5.6–8.6)	4.9 (3.4–7.0)	
Residence				0.219
Rural	24.9 (not reported)	25.1 (23.1–27.1)	24.5 (19.8–29.9)	
Urban	75.1 (not reported)	75.0 (72.9–76.9)	75.5 (70.1–80.2)	
Anthropometric				
BMI, mean (95%CI), kg/m^2^	26.5 (26.1–26.9)	25.4 (25.1–25.7)	29.2 (28.7–29.7)	0.001
NC, cm	37.6 (37.3–37.9)	36.7 (36.2–37.2)	39.9 (39.7–40.2)	0.001
Medical comorbidities				
Diabetes	6.6 (5.4–8.0)	3.4 (2.3–4.9)	14.6 (13.0–16.4)	0.001
Cardiovascular diseases	6.7 (5.5–8.2)	3.3 (2.3–4.6)	15.3 (13.7–17.0)	0.001
Hypertension	13.5 (12.7–14.4)	3.5 (2.8–4.4)	38.5 (37.3–39.6)	0.001
Sleep-related comorbidities				
RLS	14.6 (10.5–19.9)	11.2 (7.7–16.1)	23.0 (17.9–29.0)	0.001
Insomnia	60.9 (49.8–71.0)	56.7 (45.5–67.3)	71.3 (61.3–79.6)	0.001
Night sleep duration	7.0 (6.8–7.2)	7.1 (7.0–7.3)	6.7 (6.5–6.9)	0.001

Data are presented as % (95%CI); OSA, obstructive sleep apnea; BMI, body mass index; NC, neck circumference; RLS, restless legs syndrome.

^a^People who cannot attend school at the proper time, upon their willingness, can attend educational courses for adults called Nehzat.

Among 3198 individuals, 28.7% (CI 95%: 26.8–30.6) were identified as being at high risk for OSA. A history of cardiovascular disease (CVD), pulmonary, or allergy illnesses was reported by 15.2%, 3.8%, and 3.4% of the high-risk group, respectively. A history of using hypnotic, hypertensive, and neurologic medications was reported by 10.4%, 31.6%, and 8.2% of participants at high risk for OSA, respectively.

Regarding the self-reported items on STOP-BANG questionnaire, self-reported loud snoring, daytime fatigue, interrupted breathing, hypertension, and observed snoring by a partner, were 26%, 16.4%, 13.8%, 14.4%, and 33.2%, respectively. All aforementioned self-reported variables were reported more in the group with a high risk for OSA than the group without OSA (loud snoring: 57.5% vs. 6.06%, daytime fatigue: 20.34% vs. 13.96%, interrupted breathing: 29.84% vs. 3.73%, hypertension: 32.47% vs. 2.9%, observed snoring by a partner: 36.09% vs. 31.28%, see Table S2 in the supplemental material).

There was a significant difference among the participants with and without a high risk for OSA regarding their sex, age, marital status, education, history of diabetes, BMI, NC, sleep duration, RLS, and insomnia (all *p*-values < 0.001). Participants with a high risk for OSA were significantly older (50.8 vs. 35.4 years old), more likely to be married (80% vs. 67.2%), more diabetic (14.6% vs. 3.4%), less educated (high-school and higher: 59.1% vs. 80.6%), had a higher BMI (29.2 vs. 25.4 kg/m^2^), a greater NC (39.9 vs. 36.7 cm), a shorter sleep duration (6.7 vs. 7.1 h), and more likely to have insomnia (71.3% vs. 56.7%) and RLS (23% vs. 11.2%) than those without high-risk for OSA (Table 1). The male/female ratio was significantly higher in the high-risk group (males: 69.7% vs. 42.1%).

According to adjusted multiple logistic regression, OSA was significantly predicted by age, male sex, BMI, NC, RLS, and insomnia. The strongest predictor was the male sex, with an odds ratio of 3.84, followed by insomnia (OR: 2.32) and restless legs syndrome (OR: 2.03) (Table 2).

**Table 2:** Adjusted multiple logistic regression model for OSA predictors.

Variable^a^	Category	Odds ratio	95% CI	*p*-value
Demographic				
Age		1.09	1.08–1.09	0.001
Sex	Female	1		
	Male	3.84	3.24–4.56	0.001
Marital status	Single	1		
	Married	0.70	0.50–0.97	0.037
	Divorced	1.18	0.58–2.39	0.590
	Widow	0.59	0.41–0.85	0.011
Anthropometric				
BMI		1.13	1.10–1.17	0.001
NC		1.30	1.24–1.36	0.001
Medical comorbidities				
Diabetes	No	1		
	Yes	1.61	1.01–2.56	0.045
Sleep-related comorbidities				
RLS	No	1		
	Yes	2.03	1.78–2.31	0.001
ISI	No	1		
	Yes	2.32	1.94–2.78	0.001

OSA, obstructive sleep apnea; BMI, body mass index; NC, neck circumference; RLS, restless legs syndrome; ISI, insomnia severity index.

^a^Other variables were excluded in the final model of logistic regression because they have a *p*-value > 0.2.

### OSA clinical phenotypes

**Figure 1:**
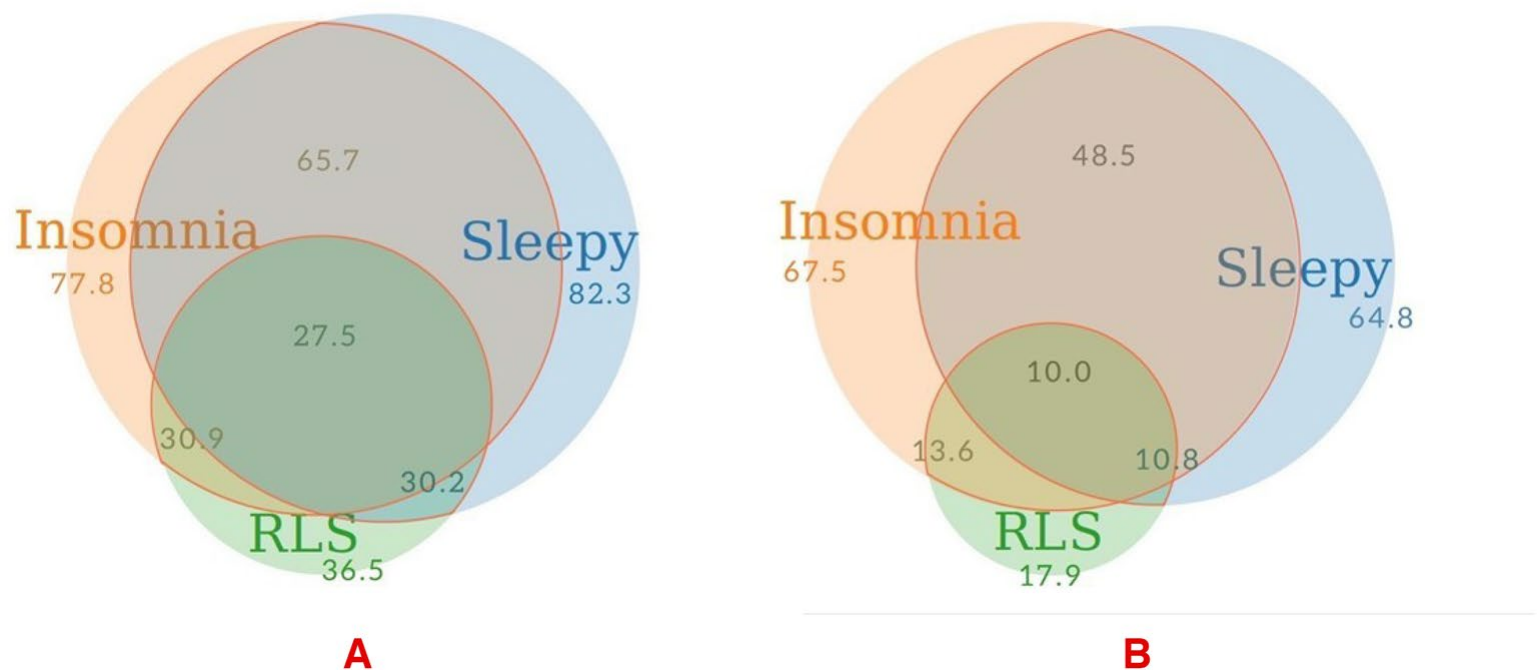
Distribution of clinical and overlapping phenotypes in (**A**). Women with OSA and (**B**). Men with OSA. The “meta-chart” app was used to illustrate proportional Venn diagrams, available at https://www.meta-chart.com.

**Figure 2:**
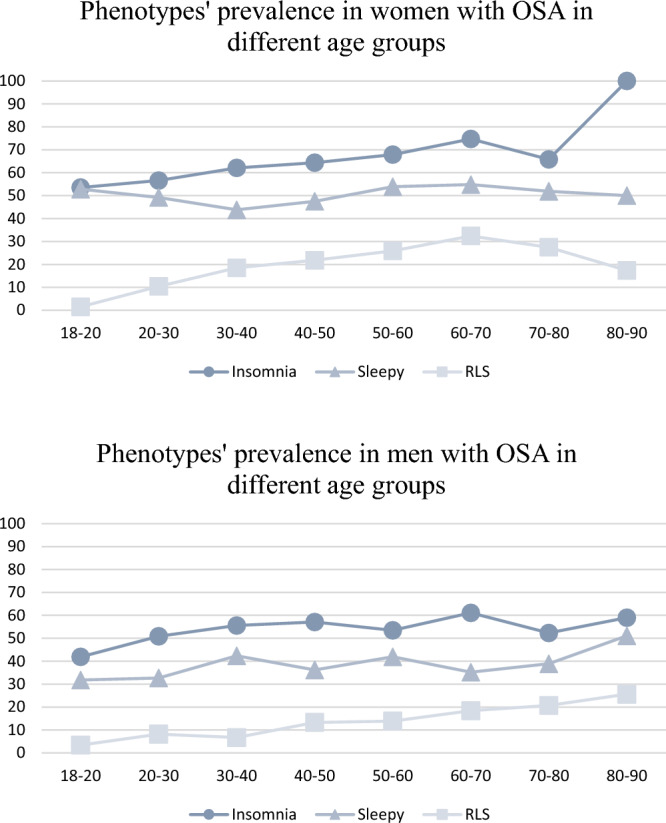
Age trend of OSA phenotypes in women and men with OSA.

In the total population with a high risk for OSA, the “sleepy” phenotype was the most frequent phenotype (82.3%) in women, and “insomnia” was the most common phenotype (67.5%) in men. “RLS” was the least common phenotype in both men and women with a high-risk for OSA. The most common overlapping phenotypes were “sleepy” and “insomnia” together in both men and women. Phenotypes overlapped more in women than men. 27.5% of women reported all 3 phenotypes at the same time while this was 10% in men (Figs. 1, 2, and Table S3 in the supplemental material).

#### Sleepy phenotype

Among women with a high-risk for OSA, those with a “sleepy” phenotype were less likely to have a history of hypertension (61.5% vs. 85%, *p*: 0.003). Among men with a high-risk for OSA, those with a “sleepy” phenotype were significantly younger (45.7 years old vs. 52.5 years old, *p*: 0.001), more likely to be single (16.8% vs. 13%, p: 0.014), less obese (26.5% vs. 34%, *p*: 0.012), with lower NC (40.5 cm vs. 41.1 cm, *p*: 0.011), and less likely to have a history of hypertension (22.4% vs. 35%, *p*: 0.001), Table 3.

**Table 3:** Factors associated with different clinical phenotypes of participants with OSA.

	Phenotype variable	Sleepy		Insomnia		RLS	
		Value	p^a^	Value	p^a^	Value	p^a^
Women	Demographic						
	Age, mean (95% CI), years	56.2 (54.7–57.6)	0.095	57.6 (56.3–59.0)	0.383	57.5 (56.5–58.6)	0.598
	Marital status		0.692		0.303		0.643
	Single	2.8 (1.0–7.4)		0.8 (0.4–1.8)		0.3 (0.04–2.5)	
	Married	77.4 (74.8–79.8)		79.2 (75.6–82.4)		78.7 (76.6–80.6)	
	Divorced	1.3 (0.6–2.6)		0.9 (0.3–2.1)		1.2 (0.7–2)	
	Widow	18.5 (14.9–22.8)		19.1 (15.3–23.7)		19.8 (17.8–21.8)	
	Residence		0.675		0.608		0.001
	Rural	22.2 (19.0–25.8)		22.4 (17.0–28.9)		13.0 (9–18.5)	
	Urban	77.8 (74.2–81.0)		77.6 (71.1–82.9)		87.0 (81.5–90.5)	
	Education		0.414		0.132		0.522
	Illiterate	17.2 (9.6–29.0)		20.9 (8.5–42.9)		16.4 (13.1-20.5)	
	Nehzat^b^	9.2 (5.9–14)		9.8 (5.6–16.7)		13.4 (11.1-16.0)	
	Primary school	34.4 (25.9–44.0)		32.0 (19.9–47.2)		33.9 (27.0-42.6)	
	Highschool/Diploma	22.9 (19.4–27.0)		23.6 (18.7–29.3)		23.2 (19.4-27.4)	
	Associate degree/Bachelor	13.3 (11.7–15.1)		11.2 (9.5–13.1)		10.7 (4.8-22.2)	
	Master and higher	3.0 (1.5–5.6)		2.5 (1.2–4.9)		2.4 (1.5-3.9)	
	Anthropometric						
	BMI		0.104		0.185		0.605
	Underweight	0		0		0	
	Healthy weight	14.0 (8.8–21.7)		16.2 (10.3–24.5)		13.4 (9.2–19)	
	Overweight	28.2 (25.5-31.0)		24.7 (22.0–27.5)		22.1 (19.4–26.7)	
	Obesity class 1	32.4 (29.6–35.3)		32.3 (29.6–35.1)		34.1 (30.4–38.1)	
	Obesity class 2	19.5 (15.2–24.8)		20.6 (17.2–24.4)		22.8 (19.4–26.7)	
	Obesity class 3	5.9 (5.1–6.8)		6.3 (3.7–10.7)		7.6 (5.2–10.9)	
	NC, cm	37.9 (37.5–38.4)	0.106	38.0 (37.5–38.4)	0.473	38.0 (37.8–38.1)	0.080
	Medical comorbidities						
	Diabetes	25.6 (22.1–29.4)	0.500	26.7 (23.4–30.2)	0.577	26.9 (23.7–30.2)	0.423
	Hypertension	61.5 (56.6–66.3)	0.003	64.4 (51.7–75.3)	0.235	64.4 (57.2–71.0)	0.892
	CVD	23.2 (17.4–30.2)	0.332	23.3 (17.2–30.8)	0.124	36.2 (32.6–39.8)	0.001
	Night sleep duration, h	6.8 (6.7–7.0)	0.922	6.7 (6.5–6.9)	0.001	6.6 (6.4–6.8)	0.034
Men	Demographic						
	Age, mean (95% CI), years	45.7 (44.8–46.6)	0.001	47.6 (46.6–48.6)	0.326	51.3 (45.8–56.9)	0.033
	Marital status		0.014		0.057		0.079
	Single	16.8 (11.6–23.8)		17.2 (14.5–20.4)		8.2 (4.1–15.7)	
	Married	79.1 (71.5–85.0)		78.8 (74.7–82.3)		85.7 (76.1–91.9)	
	Divorced	2.1 (1.3–3.4)		2.1 (1.3–3.4)		2.7 (1.5–4.7)	
	Widow	2.0 (1.0–3.9)		1.9 (0.9–3.8)		3.4 (1.9–5.9)	
	Residence		0.668		0.033		0.907
	Rural	25.4 (15.4–38.8)		23.3 (12.8–38.6)		24.9 (14.7–38.9)	
	Urban	74.6 (61.2–84.6)		76.7 (61.4–87.2)		75.1 (61.1–85.3)	
	Education		0.345		0.081		0.001
	Illiterate	5.3 (3.3–8.4)		4.9 (2.8–8.2)		7.1 (1.4–28.4)	
	Nehzat^b^	1.2 (0.4–3.2)		1.7 (0.9–2.9)		5.0 (3.1–7.8)	
	Primary school	21.2 (18.7–24.0)		21.6 (19.3–24.1)		29.9 (25.1–35.1)	
	Highschool/Diploma	36.8 (35.2–38.4)		32.6 (27.4–38.4)		30.5 (23.7–38.3)	
	Associate degree/Bachelor	27.9 (21.9–34.7)		32.1 (28.3–36.1)		22.0 (16.5–28.8)	
	Master and higher	7.6 (4.5–12.7)		7.1 (4.0–12.5)		5.5 (3.9–7.6)	
	Anthropometric						
	BMI		0.012		0.315		0.215
	Underweight	1.3 (0.7–2.4)		0.7 (0.2–2.1)		2.3 (0.5–10.6)	
	Healthy weight	23.9 (17.6–31.7)		23.9 (16.1–33.8)		24.4 (14.5–37.9)	
	Overweight	48.3 (43.1–53.5)		46.6 (41.7–51.7)		38.6 (33.8–43.7)	
	Obesity class 1	19.3 (16.4–22.5)		22.0 (18.5-25.9)		27.1 (20.0–35.6)	
	Obesity class 2	4.7 (3.6–6.1)		5.3 (4.4–6.4)		3.6 (2.0–6.4)	
	Obesity class 3	2.5 (1.8–3.4)		1.5 (0.9–2.6)		4.0 (2.4–6.6)	
	NC, cm	40.5 (40.0–41.1)	0.011	40.7 (40.1–41.4)	0.955	41.1 (40.6–41.6)	0.812
	Medical comorbidities						
	Diabetes	9.7 (8.8–10.6)	0.872	9.6 (8.6–10.8)	0.968	14.0 (10.7–18.2)	0.323
	Hypertension	22.4 (19.0–26.1)	0.001	28.4 (25.4–31.5)	0.437	40.5 (35.2–46.2)	0.001
	CVD	12.0 (10.3–14.0)	0.712	16.4 (13.8–19.4)	0.002	20.6 (17.4–24.1)	0.003
	Night sleep duration, h	6.6 (6.3–6.8)	0.281	6.4 (6.2–6.7)	0.001	6.5 (6.3–6.8)	0.712

Data are presented as mean (95% CI) and prevalence percent (95% CI); OSA, obstructive sleep apnea; RLS, restless legs syndrome; BMI, body mass index; cm, centimeter; h, hour; NC, neck circumference; CVD, cardiovascular diseases.

^a^*p*-value of comparison between participants with the mentioned phenotype and participants without the mentioned phenotype.

^b^People who cannot attend school at the proper time, upon their willingness, can attend educational courses for adults called Nehzat.

#### Insomnia phenotype

In the women with a high-risk for OSA, the “insomnia” phenotype was only associated with shorter sleep duration (6.7 h vs. 7.3 h, *p*: 0.001). In the men with a high-risk for OSA, “insomnia” phenotype was significantly associated with shorter sleep duration (6.4 h vs. 7.0 h, *p*: 0.001), greater frequency of CVD history (16.4% vs. 4.6%, *p*:0.002), and urban residence (76.7% vs. 69.6%, *p*: 0.033), Table 3.

#### RLS phenotype

The “RLS” phenotype was associated with shorter sleep duration (6.6 h vs. 7.1 h, *p*: 0.034) and a greater prevalence of CVD history (36.2% vs. 13%, *p*: 0.001) in women who were at high risk for OSA. Among males with a high-risk for OSA, those with “RLS” phenotype were older (51.3 years old vs. 47.5 years old, *p*: 0.033), less educated (illiterate and primary school: 28.2% vs. 37%, *p*: 0.001), and had a higher prevalence of hypertension (40.5% vs. 23.9%, *p*: 0.001) and CVD history (20.6% vs. 10.2%, *p*: 0.003), Table 3.

## Discussion

We found that 28.7% of the participants were classified as high-risk for OSA. The most prevalent clinical OSA phenotypes were “sleepy” in women and “insomnia” in men. Among demographic and anthropometric characteristics, age, male sex, BMI, NC, and sleep-related comorbidities, RLS and insomnia were significant OSA predictors.

This study provides more reliable information on OSA prevalence in Iran, albeit related studies in the region yielded inconsistent results from 5 to 41%. Different screening tools, non-cluster sampling methods, different local population settings, and study types could represent this discrepancy^4–6^.

In Asia, India and Turkey have indicated a prevalence of 13.7% and 25%, respectively, using the Berlin questionnaire, which is considered less accurate than the STOP-BANG^20–22^. Moreover, Iran is among the Asian countries with the most significant prevalence of OSA, that could be attributable to Iran's high obesity prevalence and body mass index^23^. However, we did not notice an averagely high BMI in our research, and the prevalence of OSA was equivalent to that of other Asian nations. It should be noted that BMI is not the only factor that affects OSA risk; further research is required on other contributing variables such as craniofacial anatomy^24^.

The current study showed a positive relationship between OSA and age, male sex, BMI, and NC. These were identified as significant OSA predictors using a multivariate logistic regression model. In agreement with our findings, numerous studies have also identified these variables as reliable OSA predictors^25–29^.

The mean BMI in the group with a high-risk for OSA was significantly higher, and BMI was one of the independent predictors of OSA. Consistent with this, Mergen et al.^30^ found a significant association between BMI and the prevalence of OSA. Although we identified a relationship between BMI and OSA, there were not many obese individuals in our sample. This aligns with the data from Djalalinia et al.’s systematic review, which highlighted an average age-standardized BMI of 25.9 for men and 27.9 for women in Iran^31^. So, there is need for further research on OSA clinical and pathophysiological phenotypes and the fact that not all OSAs are associated with obesity.

Regarding sleep-related co-morbidities, RLS and insomnia were the key contributors to OSA. Krell et al.^32^ assessed the prevalence of insomnia in patients with OSA; they found that 33.4% of the population in their sample reported having trouble sleeping, which is less than our finding. The study by Podlipnik et al.^26^ found an association between OSA and RLS, and the prevalence of RLS in the patients with OSA was 71%; which is higher than our study, this could be due to different diagnostic method of OSA in the Podlipnik study. Our findings are most comparable to those of Benedikstdottir et al.^33^, who examined a cohort of newly diagnosed patients in Iceland and discovered that the prevalence of RLS was 23.3% in men with OSA and 35.8% in women with OSA.

In agreement with Massango et al.^34^, we observed that about one-third of the group with a high risk for OSA had hypertension. Several studies have found hypertension as a predictor of OSA^23,35^.

We defined the phenotypes “sleepy,” “insomnia,” and “RLS” for individuals who had STOP-BANG ≥ 3. The most frequent phenotype among men was “insomnia,” whereas the most prevalent phenotype among women was “sleepy.” “Insomnia” is indicated as the most prevalent phenotype in the European Sleep Apnea Database, consisting of around 70% males, consistent with our findings^36^. However, “excessive daytime sleepiness” was the most common phenotype according to Ye et al.^7^, and it was also the most common phenotype in our whole sample and among women. Still, our study’s ratio was higher than theirs (more than 70% vs. 42.6%), which may be due to using different tools for measuring sleepiness (a question adopted from the STOP-BANG questionnaire on daytime in the present study vs. ESS questionnaire to define the “excessive daytime sleepiness” phenotype in Ye et al.^7^ study). While this study’s approach of using a single question to define the sleepy phenotype is a limitation, the findings can still provide valuable insights in the studied population. Future studies should consider a more comprehensive approach to identify sleepy phenotype and explore its clinical implications.

Our results indicated that OSA clinical phenotypes are more likely to overlap in females than males (Fig. 1). This could be due to the higher prevalence of all phenotypes in women. To date, there are not many studies on overlapping OSA clinical phenotypes; however the overlapping phenotype “EDS-insomnia” was also more common in women than in men (27.2% vs. 24.1%) in a study where the authors identified two clinical phenotypes: “EDS” and “insomnia”. They found that females had a higher prevalence of all traits than men^36^. Physicians must consider the much-increased occurrence of overlapping characteristics in female patients while treating OSA in these individuals. Considering phenotypic differences between men and women, particularly the overlapping phenotypes, future clinical trials would yield better results by examining women and men in separate trial groups, doing a phenotype-based approach trial, and taking overlapping phenotypes into account.

“Insomnia” was associated with shorter sleep duration in men and women with high-risk for OSA. It was associated with urban residency and a history of CVD in males. In agreement with our findings, Anttalainen et al.^36^ and Saaresranta et al.^8^ have indicated that “insomnia” is associated with CVD comorbidity, and CVD is more common in the “insomnia” phenotype than the “sleepy” phenotype. We found that the “RLS” phenotype was associated with the history of CVD, which is consistent with previous studies on this issue^37–39^.

Our study’s strength was that participants originated from different regions of country and were sampled using the door-to-door approach. By providing the workers that collected the data with rigorous training and three quality checkpoints, we also improved the sample quality. We had a closed sex-stratified look into OSA clinical phenotypes and overlapped phenotypes. Another aspect of our study was that, unlike prior studies, we did not treat “RLS” and “insomnia” as a single phenotype. A further aspect of our study was the nationwide promotion of sleep health awareness by providing the residents with information on sleep health, sleep disorders, and treatments through pamphlets and trained interviewers (see Fig. S1 in the supplemental material).

The sampling of two further provinces was not accomplished regarding the COVID-19 pandemic. A second step was planned in which individuals with a high risk for OSA would be examined by a sleep specialist and, if consented, undergo polysomnography. Unfortunately, regarding the COVID-19 pandemic, we could not perform the second step.

Another limitation of our research was the single question to characterize the sleepy phenotype. A lengthier questionnaire such as Epworth Sleepiness Scale (ESS) could cause participants’ tiredness. While this decision may limit the extent to which we can fully characterize the phenotype of sleepy patients with OSA, it was necessary to achieve our primary objective of estimating OSA prevalence in the Iranian population.

### Future directions

While our study benefits from a robust methodology, including a large sample size, clustering sampling method, and complex survey analysis, it is important to note that OSA screening relied on questionnaires. Future research might combine the advantages of large-scale surveys with the diagnostic accuracy of techniques like polysomnography for more definitive results to increase accuracy. Moreover, based on the distribution of different OSA clinical phenotypes, further studies on the association of these clinical phenotypes with pathophysiological phenotypes are required.

We recommend developing a national plan for OSA awareness, prevention, diagnosis, and management strategies. Promoting sleep health across the community and incorporating sleep health into the national health agenda by providing authorities with national data on the issue and the cost of OSA, which is important for health and insurance stockholders, appears to be necessary.

## Conclusions

Approximately a third of Iranians were at high risk for OSA, and three different and numerous overlapping OSA phenotypes were found. Therefore, implementing national health initiatives for OSA is crucial. To better identify the burden of OSA, sex-stratified trial groups, phenotype-based strategies, and study of overlapping phenotypes are recommended for the future.

### Supplementary Information

Supplementary Information.

## Data Availability

Datasets are not publicly available but are accessible upon reasonable request from the corresponding author.
