# Supplementary material for: A nationwide study on the prevalence and contributing factors of obstructive sleep apnea in Iran
Source: Sci Rep. 2023 Oct 17;13:17649. doi: 10.1038/s41598-023-44229-w (PMC10582253; doi:10.1038/s41598-023-44229-w)
Supplement: Supplementary file 1 — Supplementary Information. [file 41598_2023_44229_MOESM1_ESM.pdf]

Table S1. Sample size calculation of provinces

Geographic Regions	Random Selected Province	Population of the province	Province Weight	Province Sample size	Urban Population sample size	Rural population sample size	Required number of PSGs/province
Northeast	Khorasan Razavi	6434501	0.133337	680.0202	510.0151	170.005	238.0071
	Mazandaran	3,283,582	0.068043	347.0202	260.2652	86.75505	121.4571
Northwest	West Azerbaijan	3265219	0.067662658	345.079554	258.8096658	86.26988861	120.7778441
Southeast	Hormozgan	1738234	0.03602	183.7025	137.7769	45.92563	64.29589
	Kerman	3164718	0.06558	334.4583	250.8437	83.61457	117.0604
Southwest	Fars	4851274	0.100529	512.6993	384.5245	128.1748	179.4448
	Khuzestan	4710509	0.097612	497.8228	373.3671	124.4557	174.238
Center	Yazd	1138533	0.023592956	120.324076	90.24305729	30.0810191	42.11342673
	Tehran	13267637	0.274935	1402.169	1051.627	350.5424	490.7593

Table S2. Distribution of self-reported items of the STOP-BANG questionnaire

**Variable**		**Total**	**OSA**	
			**No**	**Yes**
Loud Snoring	No	74.0 (71.9-76.0)	93.9 (92.2-95.3)	42.5 (34.6-50.8)
	Yes	26.0 (23.9-28.1)	6.1 (4.7-7.8)	57.5 (49.2-65.4)
Daytime sleepiness and fatigue	No	83.6 (81.8-85.2)	86.0 (83.0-88.6)	79.7 (77.5-81.7)
	Yes	16.4 (14.8-18.2)	14.0 (11.4-17.0)	20.3 (18.3-22.5)
Interrupted breathing	No	86.2 (84.1-88)	96.3 (94.4-97.5)	70.2 (66.5-73.6)
	Yes	13.8 (12.0-15.9)	3.7 (2.5-5.6)	29.8 (26.4-33.5)
Hypertension	No	85.7 (84.1-87.1)	97.1 (94.5-98.5)	67.5 (66.8-68.2)
	Yes	14.3 (12.9-15.9)	2.9 (1.52-5.47)	32.5 (31.8-33.2)
Observed snoring	No	66.9 (62.5-70.9)	68.7 (60.6-75.8)	63.9 (61.1-66.6)
	Yes	33.1 (29.1-37.5)	31.3 (24.2-39.4)	36.1 (33.4-38.9)
Self-reported snoring	No	62.7 (59.9-65.4)	84.7 (79.8-88.5)	28.0 (25.3-30.7)
	Yes	37.3 (34.6-40.1)	15.3 (11.5-20.2)	72.0 (69.2-74.7)

Numbers are presented as % (95% confidence interval);

Table S3. Phenotypes' prevalence in participants with OSA

Phenotype	Men with OSA	Women with OSA
Sleepy	64.8 (57.92 -71.19)	82.3 (77.63-86.10)
Insomnia	67.5 (57.89-75.76)	77.8 (73.72-81.32)
RLS	17.9 (14.39- 22.01)	36.5 (25.78-48.78)
Sleepy and RLS	10.8 (8.03-14.43)	30.2 (21.23-41.06)
Sleepy and Insomnia	48.5 (41.53- 55.54)	65.7 (59.64 - 71.29)
RLS and Insomnia	13.6 (9.73-18.81)	30.9 (23.00-39.99)
Sleepy and RLS and Insomnia	10.0 (7.56-13.16)	27.5 (19.85-36.71)

Kerman University of Medical Sciences
Deputy of Health

SNORING

Risks & Solutions

Occupational Sleep Research Center
Baharloo Hospital
Tel. +98 21 55460184
osrc.tums.ac.ir

Questionnaire	Yes	No
Do you often feel tired, fatigued, or sleepy during the daytime?		
Do you snore loudly (louder than talking or loud enough to be heard through closed doors)?		
Has anyone observed you stop breathing during your sleep?		
Do you have or are you being treated for high blood pressure?		
Do you have a BMI of more than 35 kg/m2?		
Are you over 50 years old?		
Do you have a neck circumference >16 inches (40cm)?		
Gender: male?		

For more information contact "Occupational Sleep Research Center"

Address.
Baharloo Hospital, Behdari street,
Rahahan Square, Tehran

Tel. +98 21 55460184
Osrc.tums.ac.ir

Treatment

Most sleep breathing disorders are treatable, and according to the type and severity of the disease, the type of treatment is different. The type of treatment is determined based on the polysomnography (sleep test) results.

Weight loss, surgery, continuous positive airway pressure (CPAP), and the use of intraoral devices are all effective treatments.

Clearly, without polysomnography, treatment can be ineffective.

Attention

Surgical procedures are recommended based on PSG results.

The following can be effective in reducing snoring and stop breathing while sleeping"

Weight Loss
Side sleeping
Avoid alcohol and sleeping pills
Good sleep hygiene

How to use the sleep apnea questionnaire?

If you replied "yes" to three or more of the questions, you are at risk for sleep apnea. The greater the number of yes responses, the greater the risk and severity.

In this case, it is strongly recommended to undergo a polysomnography test (sleep test) at a reputable center under the supervision of a sleep specialist.

How to diagnose dangerous snoring?

Signs of a dangerous condition:

-Snoring with daytime sleepiness
-Snoring with hypertension
-Snoring with a breathing stop, observed by partner or roommate

If any of the above symptoms are present, the patient should be referred to a sleep clinic as soon as possible and undergo a **polysomnography (sleep test).**

How is polysomnography performed?

The patient is admitted to a room like a sleeping room, and the following items are assessed during the sleep test:

Electroencephalography
Electromyography of the face and lower limbs
Oronasal flow
Respiratory movements of the chest and abdomen
Blood oxygen saturation
Body position
Snoring sounds

This type of snoring with disruptions in breathing (apnea) indicates a sleep problem known as **"obstructive sleep apnea syndrome."**

Patients who snore are usually men, older, and obese.

Snoring can be caused by the relaxation of the muscles in this region during sleep and the narrowing of the airways.

Why snoring could be dangerous?

Snoring could be a symptom of obstructive sleep apnea. 5-10% of people with snoring have apnea.

These people are at risk of heart attack, stroke, hypertension, diabetes mellitus, decreased learning ability, excessive sleepiness during the day, impotency, and **car accidents**.

Snoring
Risks and Solutions

Everyone probably knows at least one person who snores You may also know someone who snores! It could be a relative, a spouse, or a friend.

Some people consider snoring as a simple or even funny condition. However, snoring could be a symptom of a serious medical condition known as **obstructive sleep apnea syndrome (stop of breathing during sleep)**; so this could not be considered a non-important or funny condition.

About 30-40% of adults snore while sleeping. It is more common in **men**, although **after menopause**, the prevalence increases due to hormonal changes.
Anatomical, genetic, and medications can all contribute to or worsen snoring.

Most people who snore loudly experience periods of upper airway collapse and interruption of breathing during sleep.

Figure S1. An example of the educational pamphlet of the Kerman University of Medical Sciences
